# Identification and Characterization of Two Novel Viruses in Ocular Infections in Reindeer

**DOI:** 10.1371/journal.pone.0069711

**Published:** 2013-07-16

**Authors:** Saskia L. Smits, Claudia M. E. Schapendonk, Marije van Leeuwen, Thijs Kuiken, Rogier Bodewes, V. Stalin Raj, Bart L. Haagmans, Carlos G. das Neves, Morten Tryland, Albert D. M. E. Osterhaus

**Affiliations:** 1 ViroClinics BioSciences BV, Rotterdam, The Netherlands; 2 Department of Viroscience, Erasmus Medical Center, Rotterdam, The Netherlands; 3 Section of Arctic Veterinary Medicine, Department of Food Safety and Infection Biology, the Norwegian School of Veterinary Science, Tromsø, Norway; 4 Section of Veterinary Public Health, Department of Health Surveillance, Norwegian Veterinary Institute, Oslo, Norway; 5 Genøk—Centre for Biosafety, Tromsø, Norway; Saint Louis University, United States of America

## Abstract

A thorough understanding of virus diversity in wildlife provides epidemiological baseline information about pathogens. In this study, eye swab samples were obtained from semi-domesticated reindeer ( *Rangifer* *tarandus* *tarandus*) in Norway during an outbreak of infectious eye disease, possibly a very early stage of infectious keratoconjunctivitis (IKC). Large scale molecular virus screening, based on host nucleic acid depletion, sequence-independent amplification and next-generation sequencing of partially purified viral nucleic acid, revealed the presence of a new papillomavirus in 2 out of 8 eye swab samples and a new betaherpesvirus in 3 out of 8 eye swab samples collected from animals with clinical signs and not in similar samples in 9 animals without clinical signs. Whether either virus was responsible for causing the clinical signs or in any respect was associated to the disease condition remains to be determined.

## Introduction

Infectious keratoconjunctivitis (IKC), a multifactorial eye disease with high morbidity, is a highly contagious ocular infection in domestic animals. IKC symptoms range from subclinical or mild to severe. Initial signs are photophobia, blepharospasm, and epiphora; later, the ocular discharge may become mucopurulent. Subsequently, more severe cases are characterized by increased lacrimation, edema of the conjunctiva and the periorbital region, and opacity and ulceration of the cornea, eventually resulting in blindness. IKC causes significant reductions in cattle industry production and is thought to be caused by infectious agents such as *Moraxella* *bovis* and *Neisseria* species, and influenced by other factors among which sunlight and the presence of dust and flies [1,2]. In red deer ( *Cervus* *elaphus* ), an alphaherpesvirus (cervid herpesvirus 1; CvHV1) is associated with IKC [3–5].

Reindeer ( *Rangifer* *tarandus* *tarandus*) are one of the most important land mammal species in the Arctic and sub-arctic ecosystems, with more than 4 million animals distributed over all Arctic countries. Apart from its ecological importance these animals also hold a special cultural, economic and historical value for several indigenous peoples. In Norway, as in Fennoscandia in general, reindeer herding is an important economical and cultural backbone for the Sami people. Norway holds approximately 250,000 semi-domesticated reindeer, and meat and hides are the most important commercial products. About 3,500 people are directly involved in this semi-nomadic animal production, seasonally moving the reindeer between summer- and winter-pastures and exploiting the natural pasture resources. Thus, the animals are free-ranging most of the year, and are only gathered and handled for tagging of calves, antiparasitic treatment and slaughter.

IKC was first reported in semi-domesticated reindeer more than a century ago [6] and cervid herpesvirus 2 (CvHV2), a member of the subfamily *Alphaherpesvirinae*, was recently identified as a potential causative agent in an outbreak of IKC in Norway in 2009 [7,8]. During this outbreak, 86% of clinically affected animals had antibodies against CvHV2 versus 42% of unaffected animals. Similar percentages were obtained by CvHV2-specific PCR in swab samples obtained from the eyes of clinically affected and unaffected animals. The ability of the swab sample to cause a cytopathic effect in cell culture increased with the severity of clinical symptoms, however in severe clinical cases, it was no longer possible to isolate the virus and secondary bacterial infections were dominant [7,8]. Latent alphaherpesvirus infections are reactivated upon stress, and it has been suggested that certain factors, such as reindeer density (number of animals per km^2^), herding, gathering and handling animals, may be important factors affecting virus reactivation and transmission of the endemic CvHV2 to new susceptible individuals in the herd, i.e. calves and young animals. These young animals are more likely than older animals to develop disease, such as IKC, because they undergo a primary infection. In most cases, the initial stages of IKC in reindeer (whitish and bluish cornea) seems to heal spontaneously, but sometimes IKC appears with a typical transmissible nature affecting many calves and young animals, and the viral infection may be followed by secondary bacterial infections that aggravate clinical symptoms [7,8].

Although IKC is regarded as multi-factorial, also for reindeer, it is not clear which infectious agents (bacteria and viruses) are involved in developing the disease, or which agents are commonly found in eyes of reindeer. Therefore, eye swab samples were taken from young reindeer calves with brownish discoloration of the fur around the medial eye angle caused by enhanced lacrimation and with pus running from the affected eye(s), before treatment with antibiotics. These signs are suggestive of a very early stage (grade 1) of IKC [8]. In addition, eye swabs were taken from apparently healthy calves from the same herd. We used next-generation sequencing to gain insight in the viral populations from the eyes of semi-domesticated reindeer with and without initial signs of eye disease.

## Materials & Methods

### Sample collection

A total of 17 eye swab samples were collected from a flock of semi-domesticated reindeer calves ( *Rangifer* *tarandus* *tarandus*) from Norway, in March 2012. Eye swabs were taken from animals with brownish discoloration of the fur around the medial eye angle caused by enhanced lacrimal production, suggestive of a very early stage (grade 1) of infectious keratoconjunctivitis with pus running from the affected eye(s) [7,8] (*n* = 8) or apparently healthy controls (*n* = 9) (Table 1).

**Table 1:** Reindeer (* Rangifer tarandus tarandus*) from Troms County, Norway, with and without clinical eye infections, sampled for virus amplification and sequencing.

**Clinically affected**	**Animal ID**	**Gender**	**Age (years)**	**No. reads**	**Xipapillomavirus PCR**	**Betaherpesvirus PCR**
Yes	1	F	1	13113	-	-
	2	M	1	14978	-	CvHV3
	4	F	1	20757	RtPV3	CvHV3
	5	M	1	12165	RtPV2	CvHV3
	6	F	3	8543	-	-
	8	F	2	20188	-	-
	11	M	1	15169	-	-
	14	F	1	8152	-	-
No	3	F	1	14656	-	-
	7	F	1	9168	-	-
	9	F	12	18517	-	-
	10	F	1	13997	-	-
	12	F	2	5276	-	-
	13	F	1	13891	-	-
	15	F	1	12556	-	-
	16	F	2	11712	-	-
	17	F	2	6238	-	-

### Ethics statement

No specific permission for this study was required as the reindeer are not an endangered or protected species and sampling was conducted prior to treatment in cooperation with local veterinarians and with approval of the reindeer herder/animal owner, to address the cause of the disease through diagnostics and to optimize treatment protocols for IKC in reindeer.

### PCR for detection of alphaherpesvirus

DNA was extracted from all swab samples using a DNA MiniPrep kit (Qiagen, Hilden, Germany). A nested pan-alphaherpesvirus PCR was conducted as described elsewhere [8], with primers amplifying a 294-bp region of the UL27 gene, a highly conserved gene among ruminant alphaherpesviruses, encoding glycoprotein B (gB). CvHV2 (strain Salla 82, Finland) was used as positive control. PCR products were separated by agarose gel electrophoresis and visualized with SYBR green (Roche Applied Biosciences, Basel, Switzerland). Amplicons of expected size were purified and sequenced as described previously [8].

### Large scale molecular virus screening

Large scale molecular virus screening, based on host nucleic acid depletion, viral nucleic acid isolation, sequence-independent amplification and next-generation sequencing with a 454 GS Junior Instrument (Roche) was performed as described previously [9,10]. More than 219,000 trimmed reads were assembled using de novo assembly and analyzed according to BLAST searches [9,10]. Sequences were classified based on the taxonomic origin of the best-hit sequence [9,10]. E values of 1.0 x 10^-3^ and 1.0 x 10^-10^ were used as cut-off value of significant virus hits for BLASTn and BLASTx searches, respectively.

### PCR amplification and Sanger sequencing

Sequence information obtained from the large scale molecular virus screening using next-generation sequencing was used to design specific primers for amplification of the papillomavirus genome RtPV2 using AmpliTaq Gold DNA polymerase (Roche), according to instructions of the manufacturer to confirm and/or extend 454-sequence reads. Primer sequences are available on request. Products were cloned and sequenced as described previously [10]. A diagnostic reindeer papillomavirus (RtPV2) PCR was developed, using primers VS702 (5’- GACACGGATGACTTTG -3’) and VS703 (5’- CCCAGTATTCCCCATC -3’) that target the L1 genome region and AmpliTaq Gold DNA polymerase (Roche), according to instructions of the manufacturer. A diagnostic betaherpesvirus (CvHV3) PCR was developed, using primers VS708 (5’- CATATGAGGTAGAAAATAAGC -3’) and VS709 (5’- AAAGTGTCAGAAGTAGGATAT -3’) that target the genome region corresponding to nucleotides 61512-61892 of human herpesvirus 7 (strain RK; GenBank AF037218; gene U40), encoding a subunit of terminase (herpesvirus core gene UL28 family; similar to HHV5 UL56) and AmpliTaq Gold DNA polymerase (Roche), according to instructions of the manufacturer.

### Genome organization and phylogenetic analysis

For genome organization analysis, putative ORFs and their corresponding amino acids were predicted using the ORF finder tool (http://www.ncbi.nlm.nih.gov/gorf/gorf.html), and similarity analysis was performed using BLAST. Multiple alignments were created using ClustalX (2.0.10) [11]. Phylogenetic analyses were carried out with Molecular Evolutionary Genetics Analysis (MEGA), version 5 [12] in a manner similar to that used in previous papilloma- and herpesvirus studies [13,14]. Phylogenetic papillomavirus trees were calculated on nucleotide sequence alignments using Maximum Likelihood method with the Kimura 2-parameter model, and bootstrap analysis was performed with 1,000 replicates. Phylogenetic herpesvirus trees were calculated using Neighbor-joining method with p-distance on nucleotide sequence alignments, and bootstrap analysis was performed with 1,000 replicates.

### Nucleotide sequence accession numbers

The nucleotide sequence of the RtPV2 whole genome and the partial L1 sequence of RtPV3 of sample 4 have been deposited in the GenBank sequence database under accession numbers: KC810012 and KC810013. The nucleotide sequence of the CvHV3 U40 gene region has been deposited in the GenBank sequence database under accession number: KC810014. Our deep-sequencing dataset was deposited at the European Nucleotide Archive under archive number PRJEB3981.

## Results

## Case report

**Figure 1:**
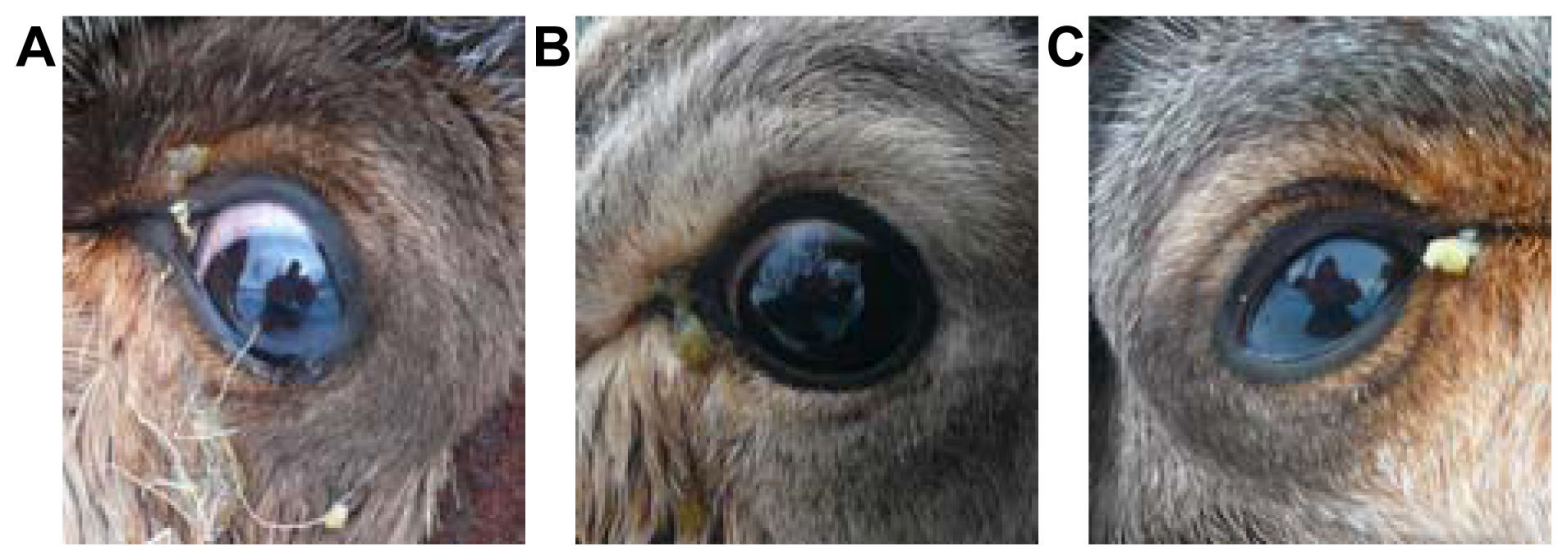
Clinical signs of eye infection in semi-domesticated reindeer during an outbreak in Norway in 2012. Photographs show animals 2 (A), 4 (B), and 5 (C) (Table 1) with a similar level of disease severity with increased lacrimation and pus running from the medial eye angle, suggestive of a very early stage of IKC [8].

The natural infectious eye disease outbreak started in Sørreisa in Troms County, Norway, in March 2012. The reindeer calves were bought by the reindeer owner to recruit live animals for his herd from Finnmark County, and transported by car for approximately 8 hours to Troms County. They were held in a fence for observation, and the outbreak started five days after arrival, with fluid running from the eyes causing discoloration of the fur of a few animals, and the condition seemed to have a transmissible appearance. At inspection one week after arrival of the animals, there were no animals having white or blue cornea, the initial signs of IKC in reindeer [8], but the brownish discoloration of the fur around the medial eye angle caused by enhanced lacrimation and pus running from the affected eye(s) suggested a very early stage of the eye infection (Figure 1; Table 1). Eight clinically affected animals and nine unaffected animals that appeared healthy were restrained for inspection and sampling prior to ocular treatment of affected animals by antibiotics (Table 1). As all animals received ocular treatment, it remains unclear if the eye disease would have developed into typical IKC if left untreated.

### PCR and large scale molecular virus screening

All animals were negative for CvHV2-specific DNA when investigated by the pan-alphaherpesvirus PCR. Large scale molecular virus screening was performed on eye swabs from clinically affected and unaffected animals. Most of the identified sequences obtained by next-generation sequencing were of eukaryotic, bacterial or unknown origin. All of the samples showed evidence for the presence of bacteriophages from the order *Caudovirales*. Eukaryotic viruses from the *Herpesviridae* and *Papillomaviridae* families were detected, some with relatively low homology on the protein level to known viruses.

### Papillomavirus

**Figure 2:**
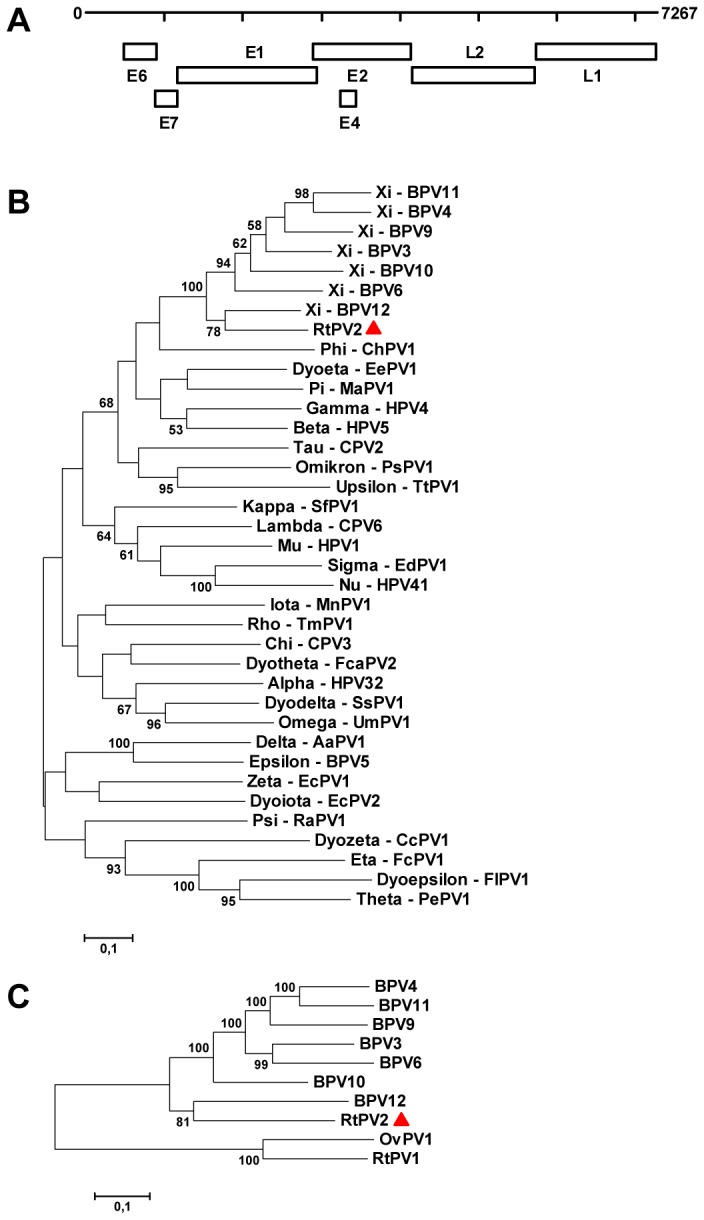
Genome organization and phylogenetic analysis of RtPV2. **A. Genome organization of RtPV2**. The line indicates the complete genome with size indication. The boxes represent open reading frames encoding early (E) and late (L) proteins). B–C. Phylogenetic trees of the L1 ORF (B) and complete genome (C) nucleotide sequences of selected representative papillomaviruses were generated using MEGA5, with the Maximum Likelihood method with Kimura-2 parameter model and 1,000 bootstrap replicates. Significant bootstrap values are shown. The different papillomavirus genera are indicated. Human papillomavirus (HPV1, HPV4, HPV5, HPV32, HPV41), V01116, NC_001457, M17463, NC_001586, NC_001354;. *Canis familiaris* papillomavirus (CPV2, CPV3, CPV6), NC_006564, NC_008297, NC_013237; European elk papillomavirus (AaPV1), NC_001524; *Sus scrofa* papillomavirus (SsPV1), NC_011280; *Francolinus* *leucoscepus* papillomavirus 1 (FlPV1), NC_013117 ; *Erinaceus* *europaeus* papillomavirus 1 (EePV1), NC_011765; *Equus caballus* papillomavirus 1 (EcPV1), NC_003748; *Equus caballus* papillomavirus 2 (EcPV2), NC_012123; *Felis* *domesticus* papillomavirus 2 (FcaPV2), EU796884; *Caretta* *caretta* papillomavirus 1 (CcPV1), NC_011530; Bovine papillomavirus (BPV3, 4, 5, 6, 9, 10, 11, 12), NC_004197, . X05817, NC_004195, AJ620208, AB331650, AB331651, AB543507, JF834523; *Fringilla* *coelebs* papillomavirus (FcPV1), NC_004068; *Mastomys natalensis* papillomavirus (MnPV1), NC_001605; Cottontail rabbit papillomavirus (SfPV1), NC_001541; *Ursus* *maritimus* papillomavirus 1 (UmPV1), NC_010739; *Capra* *hircus* papillomavirus 1 (ChPV1), NC_008032; *Phocoena* *spinipinnis* papillomavirus (PsPV1), NC_003348; Hamster oral papillomavirus (MaPV1), E15111; *Rousettus* *aegyptiacus* papillomavirus 1 (RaPV1), NC_008298; *Trichechus* *manatus* papillomavirus 1 (TmPV1), NC_006563; *Erethizon* *dorsatum* papillomavirus 1 (EdPV1), NC_006951; *Psittacus* *erithacus* timneh papillomavirus (PePV1), NC_003973; *Tursiops* *truncatus* papillomavirus 1 (TtPV1), NC_011109; *Odocoileus* *virginianus* papillomavirus 1 (OvPV1), NC_001523; *Rangifer* *tarandus* papillomavirus (RtPV1, RtPV2), AF443292, KC810012.

A full-length papillomavirus genome sequence was amplified and sequenced from an eye swab of a clinically affected reindeer (number 5), to complete and/or confirm the findings by next-generation sequencing. This papillomavirus was different from the previously identified RtPV1 from reindeer [15] and was designated *Rangifer* *tarandus* papillomavirus 2 (RtPV2). The circular genome organization of RtPV2 was typical for papillomaviruses, consisting of a long control region (LCR), and early (E6, E7, E1, E2, E4) and late regions (L1, L2) (Figure 2A). Papillomaviruses are taxonomically defined on the basis of phylogenetic distances among the L1 DNA sequences, with interpretation based on phylogeny, genome organization, biology, and pathogenicity [17,18]. Intergeneric identities range from about 43–62%, interspecies identities from about 55–71%, and intraspecies identities from about 67–88% (17,18). The RtPV2 L1 nucleotide sequence was compared to all known papillomaviruses for which a full-length L1 sequence was present in PAVE (http://pave.niaid.nih.gov/#home). The most closely related papillomaviruses to RtPV2 were bovine papillomaviruses BPV3, BPV4, BPV6, BPV9, BPV10, BPV11, and BPV12 from the genus Xipapillomavirus with 71.0-74.7% nucleotide identity in L1. A phylogenetic tree was generated for a subset of representative papillomaviruses for visibility (Figure 2B). Based on the sequence identity criteria, RtPV2 could be designated as a new papillomavirus type in the genus Xipapillomavirus. However, the fact that RtPV2 is identified in a host species other than cattle, which is in contrast to the bovine papillomaviruses comprising this genus, and that this new virus has a characteristic E6 ORF (Figure 2A), in contrast to the other Xipapillomaviruses, may suggest that this new papillomavirus from reindeer belongs to a new papillomavirus genus. This is underscored by a whole genome sequence alignment of RtPV2 with bovine Xipapillomaviruses, which showed much lower sequence identities (61.7-63.8%) across the genome and no significant bootstrap support to cluster RtPV2 with most Xipapillomaviruses, except BPV12 (Figure 2C).

To obtain insight in the prevalence of RtPV2-like viruses in reindeer eyes, a RtPV2-specific PCR targeting the L1 region of the papillomavirus genome (~500 bp) was performed on the eye swabs of the eight clinically affected animals and the nine animals that appeared healthy (Table 1). Besides the sample in which RtPV2 was identified (reindeer 5), one swab sample from an affected animal (reindeer 4) was positive for a papillomavirus sequence (RtPV3) with 71.3% nucleotide identity to RtPV2 and 68.6% identity to BPV3.

### Herpesvirus

**Figure 3:**
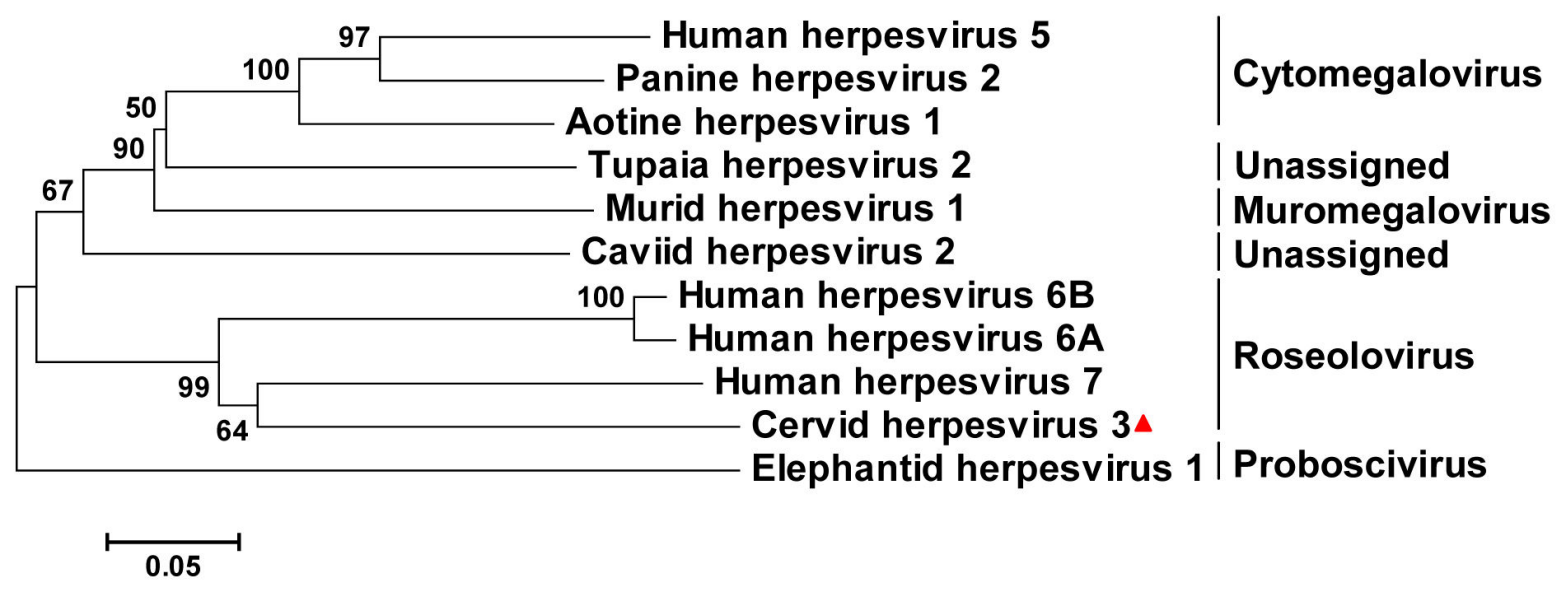
Phylogram of nucleotide sequence data for a ~400-bp genome fragment from cervid herpesvirus 3 (corresponding to nt 61499-61904 of human herpesvirus strain RK; GenBank AF037218) compared with data for the orthologous regions of selected representative mammalian herpesviruses. The phylogenetic tree was generated with MEGA5, with the neighbor joining method and the p-distance model. Bar, evolutionary distance of 0.05. Bootstrap values (1000 reiterations) are shown. The different herpesvirus genera are indicated. Human herpesvirus 5, NC_006273; Panine herpesvirus 2, AF480884; Aotine herpesvirus 1, FJ483970; Tupaia herpesvirus 2, AF281817; Murid herpesvirus 1, HE610456:. Caviid herpesvirus 2, AB592928; Human herpesvirus 6A, X83413; Human herpesvirus 6B, AF157706; Human herpesvirus 7, AF037218; Elephantid herpesvirus 1, AAF322977; Cervid herpesvirus 3, KC810014.

Using large-scale molecular virus screening, herpesvirus-like sequences were detected in an eye swab of clinically affected reindeer 2 (Table 1). Using BLASTx, these sequences showed homology to human herpesvirus 7 (HHV7), which belongs to the subfamily *Betaherpesvirinae*. Thus, this reindeer herpesvirus was different from the previously identified cervid alphaherpesviruses 1 and 2 from deer and reindeer, respectively [16] and was designated cervid herpesvirus 3 (CvHV3). A phylogenetic tree was constructed based on a ~400 bp sequence fragment of an eye swab from reindeer 2 with ~65% identity to nucleotides 61499-61904 of HHV7 strain RK (AF037218; gene U40), encoding a subunit of terminase (herpesvirus core gene UL28 family; similar to HHV5 UL56) and the corresponding sequences of representative betaherpesviruses (Figure 3). The phylogenetic tree showed that CvHV3 was most closely related to HHV7 and belonged to the genus *Roseolovirus*.

Betaherpesvirus-like sequences were also detected in eye swabs from reindeer 4 and 5 by next-generation sequencing (Table 2). Again these sequences were most closely related to members of the genus *Roseolovirus*. All the animals with CvHV3-like sequences (reindeer 2, 4 and 5) showed clinical signs of disease. To obtain insight in the prevalence of CvHV3 in reindeer eyes and its potential involvement in causing disease, a CvHV3-specific PCR targeting the U40 gene region of the herpesvirus genome from Figure 3 (~350 bp) was performed on the eye swabs of the eight clinically affected animals and the nine unaffected animals that appeared healthy (Table 1). Besides the eye swab from reindeer 2 that was previously identified to be positive for CvHV3 by next-generation sequencing, eye swabs from clinically affected animals 4 and 5 were positive for CvHV3 (100% identity). CvHv3 was not detected in eye swabs from the other five reindeer with clinical signs, or from any of the nine reindeer without clinical signs.

**Table 2:** Next generation sequencing results that yielded herpesvirus sequences from reindeer 2, 4 and 5.

**Animal ID**	**Contig size (bp)**	**No. reads**	**Reference genome**	**Gene**	**% coverage**	**% amino acid identity**
2	407	2	HHV7 (BAA13123)	U40	90	50
4	234	2	HHV7 (YP073775)	U35	84	53
5	229	8	HHV6 (NP_042921)	U28	85	46

## Discussion

In this study, early onset infectious eye disease samples, suggestive of a very early stage of IKC [8], were studied for the presence of viral infections by large scale molecular virus screening. CvHV2 sequences were not detected using random amplification of nucleic acids and a next-generation sequencing platform or by pan-alphaherpesvirus PCR in any of the animals either with or without clinical signs. This observation is seemingly in contrast to previous observations in a reindeer herd in Norway, where CvHV2 was implicated as a causative agent in IKC [8]. However, eye swabs in this study were taken at a very early stage of the eye infection with increased lacrimation suggestive of an early stage of IKC, in contrast to the previous study in which mainly later, but still initial stages of the disease were sampled, from animals with a whitish or bluish cornea, or from animals with more severe symptoms [8]. Alternatively, the infectious eye disease reported here may not have developed into IKC at all if left untreated.

A new papillomavirus, RtPV2, was identified in a clinically affected animal. Papillomaviruses are classified based on nucleotide sequence identity in the L1 ORF and their biological and pathological properties [17,18]. A papillomavirus strain is a new type if the complete genome has been sequenced and the L1 ORF shares less than 90% homology with the closest known papillomavirus type [17,18]. L1 is the most conserved region among papillomaviruses and according to the current genus classification, most papillomavirus types within a genus share more than 60% nucleotide identity in L1. Based on these criteria, RtPV2 could be designated as a new papillomavirus type in the genus *Xipapillomavirus*. However, the fact that RtPV2 is identified in a host species other than cattle, which is in contrast to the bovine papillomaviruses comprising this genus, and that this new virus has a characteristic E6 ORF, in contrast to the other Xipapillomaviruses, may suggest that this new papillomavirus from reindeer belongs to a new papillomavirus genus. The prevalence of RtPV2-like viruses in reindeer with or without clinical signs was low, with only one other animal with clinical IKC signs being positive for an RtPV2-like virus (RtPV3), which based on sequence identity in the L1 genome region to RtPV2 seems to constitute another papillomavirus type in the genus. In addition, papillomaviruses in other host species are generally not associated with conjunctivitis. Overall, it is thus unlikely that RtPV2-like viruses are involved in causing the observed disease signs. They might be involved in causing conjunctival papillomas [19,20], but in spite of the fact that skin papillomas are reported in reindeer from time to time, not much knowledge exists on papillomatosis in reindeer as a disease, or the virus(es) causing them. The impact of papillomavirus infections on reindeer in general and the specific role of RtPV2 thus has to be further addressed.

Based on phylogenetic analyses, the newly identified herpesvirus CvHV3 can be placed in the subfamily *Betaherpesvirinae*, genus *Roseolovirus*. CvHV3 is the first reported non-human herpesvirus in the genus *Roseolovirus*, which until now only contained human herpesvirus 6 and 7. The prevalence of CvHV3 in reindeer with clinical signs was 38%, whereas the virus was not detected in animals without clinical signs. Herpesviruses are known for establishing lifelong infections and for causing many different diseases, ranging from oral and genital herpes to chicken pox, shingles, lymphoma, retinitis, and carcinoma in humans. Also in animals, herpesviruses have been associated with a range of clinical conditions, such as rhinotracheitis, vaginitis, balanoposthitis, abortion, conjunctivitis, and enteritis. The human roseoloviruses 6 and 7 can cause *exanthema subitum*, whereas human herpesvirus 5, another betaherpesvirus (genus *Cytomegalovirus*), is known to cause retinitis among other diseases [21]. Thus, like CvHV2, CvHV3 may be involved in causing infectious eye disease and possibly IKC, but Koch’s postulates should be investigated to address this further [8,22–24].

(Re-) Emerging infectious diseases pose a continuous health threat to wild and domestic animals as well as to humans. It remains of utmost importance to combat newly emerging viral diseases and epidemics by obtaining a thorough understanding of the diversity of viruses in wildlife, which provides epidemiological baseline information about pathogens and may lead to early identification of newly emerging pathogens in the future [25]. The here-described discovery of a new papillomavirus and herpesvirus from reindeer is an example of the required expansion of our knowledge of the virus diversity present in wildlife, as well as of their possible impact on health and disease of these animals and the risk of transmission to and infection of production animals.
